# Phase II trial of first-line chemoradiotherapy with intensity-modulated radiation therapy followed by chemotherapy for synchronous unresectable distant metastases rectal adenocarcinoma

**DOI:** 10.1186/1748-717X-8-10

**Published:** 2013-01-07

**Authors:** Ji Zhu, Peng Lian, Fangqi Liu, Ye Xu, Junyan Xu, Zuqing Guan, Liping Liang, Minghe Wang, Sanjun Cai, Zhen Zhang

**Affiliations:** 1 Department of Radiation Oncology, Fudan University Shanghai Cancer Center, Shanghai, 200032, China; 2 Department of Colorectal Cancer, Fudan University Shanghai Cancer Center, Shanghai, 200032, China; 3 Department of Oncology, Shanghai Medical College, Fudan University, Shanghai, 200032, China; 4 Department of nuclear medicine, Fudan University Shanghai Cancer Center, Shanghai, 200032, China

**Keywords:** Rectal cancer, Synchronous unresectable distant metastases, Chemoradiotherapy, Imtensity modulated radiation therapy

## Abstract

**Aims:**

Based on the hypothesis that first-line chemoradiation followed by chemotherapy was superior for primary tumor and non-inferior for distant lesions compared to chemotherapy alone in synchronous unresectable distant metastases rectal adenocarcinoma, this study was designed to assess the efficacy and safety of this strategy.

**Materials and methods:**

Thirty two eligible patients received intensity modulated radiation therapy (45 Gy to the pelvis and a concomitant 10 Gy boost to the gross tumor), along with concurrent weekly capecitabine and oxaliplatin. Patients underwent radical surgery if all lesions were visually evaluated as resectable and received chemotherapy for a total of 6 months, whether pre- or post-operatively (definitive therapy group). The remaining patients received 6 months of consolidation chemotherapy followed by maintenance chemotherapy (non-definitive therapy group).

**Results:**

The toxicities were acceptable, with radiation-induced dermatitis around the anal verge being the most common (18.8%). Fourteen patients underwent surgical resection of the rectal tumor, with 5 (35.7%) experiencing a pathological complete response. Nine out of 14 received definitive treatment, defined as R0 resections of all visible tumors. At a median follow-up of 12 months (range, 4–23 months), 2 cases were evaluated as local failure, and the median overall survival (OS) and progression free survival (PFS) for all 32 patients were 17.5 and 12 months, respectively. OS differed significantly in the definitive and non-definitive therapy groups (p=0.045), and PFS tended to differ (p=0.274).

**Conclusion:**

It was demonstrated that the strategy of first-line chemoradiation followed by chemotherapy was effective and tolerable, especially for local control. OS and PFS were superior in patients who did than did not undergo curative therapy.

## Introduction

Synchronous distant metastases have been detected in 15%–25% of patients newly diagnosed with rectal cancer [1,2]. Surgery remains the only potentially curative modality, with complete surgical resection of all visible tumors resulting in a 5-year survival rate of 30%–40% [3-5]. However, approximately 80%–90% of newly diagnosed patients have unresectable metastatic disease [2,6,7].

Systematic chemotherapy has been used to treat patients with metastases from colorectal cancer. For example, of 1014 initially unresectable patients with colorectal liver disease treated with chemotherapy, with most receiving oxaliplatin, 138 (12.5%), evaluated as “good responders”, were able to undergo secondary hepatic resection, with a 5-year overall survival (OS) rate of 33% [5]. The intergroup N9741 trial found that the rate of curative resection after chemotherapy was 3.3%, and the median overall survival time for this group was 42.4 months [8].

To date, there is no consensus regarding the use of radiotherapy in the treatment of synchronous metastases from rectal cancer, although neoadjuvant chemoradiation has shown significant benefits in patients with stage II/III rectal cancer, including better local control and sphincter preservation [9-11]. This prospective clinical trial therefore assessed the efficacy and safety of first-line chemoradiotherapy in patients with synchronous metastases of rectal cancer; we also compared our results with historical data on patients who received first-line chemotherapy alone.

## Methods and materials

### Eligibility criteria

We enrolled patients aged 18 to 75 years with histologically confirmed, newly diagnosed, rectal adenocarcinoma, concurrent with synchronous unresectable distant metastases. The primary tumor in each patient was a locally advanced mass (cT3-T4 and/or cN+), located within 12 cm from the anal verge. Each patient had significant local symptoms, such as pain, bleeding or incomplete obstruction; a Karnofsky Performance Status ≥60; Adequate bone marrow (leukocyte count >4,000/mL, platelet count >100,000/mL), renal (creatinine clearance > 50 mL/min) and hepatic (bilirubin ≤2 mg/mL) function. Patients were excluded if they had previously received pelvic radiotherapy or chemotherapy; had previous tumors other than nonmelanoma skin cancer; or had ischemic heart disease, inflammatory bowel disease, malabsorption syndrome, peripheral neuropathy, or psychological disorders. Each patient provided written informed consent.

### Baseline evaluation

Pretreatment evaluation, performed within 2 weeks before initiation of study treatment, included a complete history and physical examination including a digital rectal examination, complete blood count, platelet count, liver and renal function tests, assays of gastrointestinal tumor markers, colonoscopy with biopsy, computed tomography (CT) of the chest and abdomen, and magnetic resonance imaging (MRI) of the pelvis, with selected patients evaluated by positron emission tomography-computed tomography (PET-CT) scanning.

### Intensity modulated radiation therapy (IMRT)

All patients were immobilized in the prone position using a belly board and underwent a non-contrast-enhanced, planning CT with 5-mm slices from the L3-L4 junction to 2 cm below the perineum. Imaging data were transferred to the PINNACLE planning system (Philips Radiation Oncology Systems, Milpitas, CA). The clinical target volume 1 (CTV1) included the gross tumor volume and the corresponding mesorectum plus 2 cm cranio-caudally [12]. The CTV2 included the CTV1 plus the entire mesorectum, the entire pre-sacral space, the internal iliac nodes and the high-risk anatomical and nodal sub-sites, based on the distance of the tumor from the anal margin. The planning target volume (PTV) was defined as the CTV with 10-mm margins superiorly and inferiorly and 8-mm margins in all other directions.the CTV plus 0.8 cm margin in all directions. Organs at risk (OARs) were contoured as follows: 1) the small intestine was defined as all intestinal loops below the sacral promontory (recto sigmoid junction excluded); 2) femoral heads were contoured from the cranial extremity to the level of the lower margin of ischial tuberosities; 3) the bladder was contoured entirely with no distinction between the wall and its content [13]. The IMRT plans were generated using the inverse planning module of PINNACLE for a 6-MV liner accelerator, with five to seven coplanar fields. The planned doses to the PTV1 and PTV2 were 55 Gy and 45 Gy, in 25 fractions, 5 times per week (Monday through Friday) for 5 weeks. The positioning and isocenter of each patient were verified on electronic portal imaging device (EPID) films for the anterior and lateral gantry positions by visually comparing the digitally reconstructed radiographs.

### Concurrent chemotherapy

Patients received capecitabine (625 mg/m2 twice daily) plus oxaliplatin (50 mg/m2 weekly) concurrent with pelvic radiation throughout the entire course of radiotherapy.

### Chemotherapy and surgery

Patients were evaluated after the completion of CRT and every two months later. If all lesions were evaluated as resectable by our weekly tumor board in six months, patients would be planed to receive a radical surgery. The sequence of primary and secondary tumors, the type of surgery (lower anterior or abdominal–perineal resection) and whether a temporary colostomy should be performed was at the discretion of each surgeon. Patients were scheduled to receive a total of 6 months of chemotherapy, whether pre- or post-operative, with Xelox (intravenous oxaliplatin 130 mg/m2 on day 1 plus oral capecitabine 1000 mg/m2 twice daily on days 1–14 every three weeks). For these patients who had no chance to receive a radical surgery, it was recommended to receive 6 months’ Xelox, followed by maintenance chemotherapy with capecitabine alone. If tumor progressed, the chemotherapy regimen would be changed to FOLFIRI regimen.

Patients at high risk of obstruction or significant bleeding underwent palliative surgery to remove the primary tumor.

### Evaluation of tumor response

Physical examinations and blood counts were performed every week during chemoradiation. Hepatic, renal function tests, computed-tomography (CT) scans of thorax and abdomen, magnetic Resonance Imaging (MRI) of pelvis were assessed at baseline and repeated every 2 months. Tumor response was assessed according to the RECIST criteria [14]. Complete response (CR) was defined as complete disappearance of all clinically assessable disease for at least 4 weeks, and partial response as a decrease of at least 30% of the sum of the products of the diameters of measurable lesions for at least 4 weeks. CT scans were done 4 weeks later to confirm a response. Stable disease was defined as a decrease of less than 30% or an increase of less than 20% of measurable lesions, and progressive disease as an increase of at least 20% of measurable lesions or the appearance of new malignant lesion(s). All CT/MRI scans were subjected to external review by at least two radiologists.

### Evaluation of toxicity

Toxicity was evaluated according to the CTC-AE (Common Terminology Criteria for Adverse Events) 3.0 criteria. Doses were adjusted or discontinued in patients with grade 3 toxicity; in general, the sequence of dose adjustment or discontinuation was oxaliplatin, capecitabine and radiotherapy, unless the adverse effect was strongly associated with a particular agent.

### Study design and endpoints

The primary end point of this trial was the objective response rate (ORR, defined as CR+PR). This is a phase II study of 32 patients to evaluate the treatment efficacy. Based on published literatures, the objective response rate is approximately 50% for patients treated with chemotherapy alone [15]. We hypothesize that the ORR of first-line CRT was 20% superior in primary tumor and no-inferior in distant lesions to CT alone. With evaluation of all 32 patients, if more than 20 and 13 cases in primary and distant tumor were evaluated as objective response, we had 85% power to reject the null hypothesis that first-line CRT followed by CT couldn’t reach the ORR of 70% for primary tumor and 50% for distant lesions. Secondary endpoints included toxicity, PCR rate and survival rates. Classified variables were described as frequencies; normal distributional continuous data as means and standard deviations; and non-normal distributional continuous data as medians. Survival time was calculated from the beginning of chemoradiotherapy (CRT) to the date of an event or last follow-up. The Kaplan-Meier method was used to estimate survival and PFS curves, and the log-rank test was used to compare the curves [16].

## Results

### Demographic and clinical features

Between January 2010 and June 2011, 32 patients were enrolled in this study (Table 1). There were 28 males and 4 females, of median age 58.5 years (range, 35–75 years). The median distance of the rectal mass from the anal verge was 5 cm (range, 3–10 cm). Nineteen patients were classified as T3 and 13 as T4; lymph node involvement was detected in all patients, with 29 of N2 status. Liver metastases were the most common distant lesions, observed in 28 patients; in addition, 26 patients had more than 3 distant lesions and 12 showed involvement of more than one distant organ.

**Table 1:** Demographic and clinical characteristics of all patients

	**No.**	**%**
Gender		
Male	28	87.5
Female	4	12.5
Age		
Median	58.5	
Range	35-75	
Distance from anal verge		
Median	5	
Range	3-10	
Initial T stage		
T3	19	59.4
T4	13	40.6
Initial N stage		
N1	3	9.4
N2	29	90.6
Initial M stage		
M1a	20	62.5
M1b	12	37.5
Distant metastases organs		
liver alone	16	50.0
lung alone	3	9.4
bone alone	1	3.1
liver and lung	10	31.3
liver and intra-abdominal	2	6.3
Total metastasis number		
1	1	3.1
2	2	6.3
3	3	9.4
>3	26	81.3
Total	32	

### Combined chemoradiotherapy

Twenty-nine patients received radiation doses of 55 Gy, the other 3 received irradiation dose ranging from 46.2 Gy to 52.8 Gy. All patients completed concurrent weekly capecitabine and at least 3 cycles of oxaliplatin during the course of CRT.

Most of the adverse events during CRT were mild (Table 2). No grade 4–5 toxicities were observed. The most common grade 3 toxicity was radiation dermatitis around the anal verge (18.8%), with 5 patients (15.6%) having grade 3 gastrointestinal toxicity.

**Table 2:** Toxicity during the course of chemoradiation

	**Grade 1**		**Grade 2**		**Grade 3**	
	**n**	**%**	**n**	**%**	**n**	**%**
Diarrhea	11	34.4	5	15.6	5	15.6
Hematologic	6	18.8	3	9.4	2	6.3
Fatigue	4	12.5	4	12.5	2	6.3
Radiation dermatitis	9	28.1	9	28.1	6	18.8
Neurosensory	1	3.1	1	3.1	0	0.0
Hand-foot syndrome	0	0	1	3.1	0	0.0

### Tumor response to CRT

All patients were evaluated for tumor response at 2 months after the beginning of CRT, including responses of the rectal mass and distant metastases. The response rate of rectal masses was 68.75% (22/32), including 4 patients with clinical complete response (cCR) and 18 with clinical partial response (cPR), and 2 showed progressive disease (PD). The response rate of distant lesions was 50% including 1 cCR and 15 cPR. Progression was observed in two patients whose primary tumor was also evaluated as PD.

### Surgery

Nine patients underwent surgical resection of all visible lesions and received definitive therapy. The other 5 patients underwent a resection of only primary tumor (Table 3). The median interval between the completion of CRT and primary tumor’s surgery was 11 weeks (range, 6–24 weeks), with 7 each undergoing APR and AR. YpT0 and ypN0 were found in 5 (35.7%) and 10 (71.4%) patients, respectively, with 5 patients (35.7%) showing a pathologic complete response (pCR). Down-staging of the primary tumor occurred in 12 patients (85.7%).

**Table 3:** Pathologic results of primary tumors

	**No.**	**%**
YpT stage		
T0	5	35.7
T2	1	7.1
T3	7	50.0
T4	1	7.1
YpN stage		
N0	10	71.4
N1	3	21.4
N2	1	7.1
Downstaging		
Yes	12	85.7
No	2	14.3
Total	14	

### Follow up

**Figure 1:**
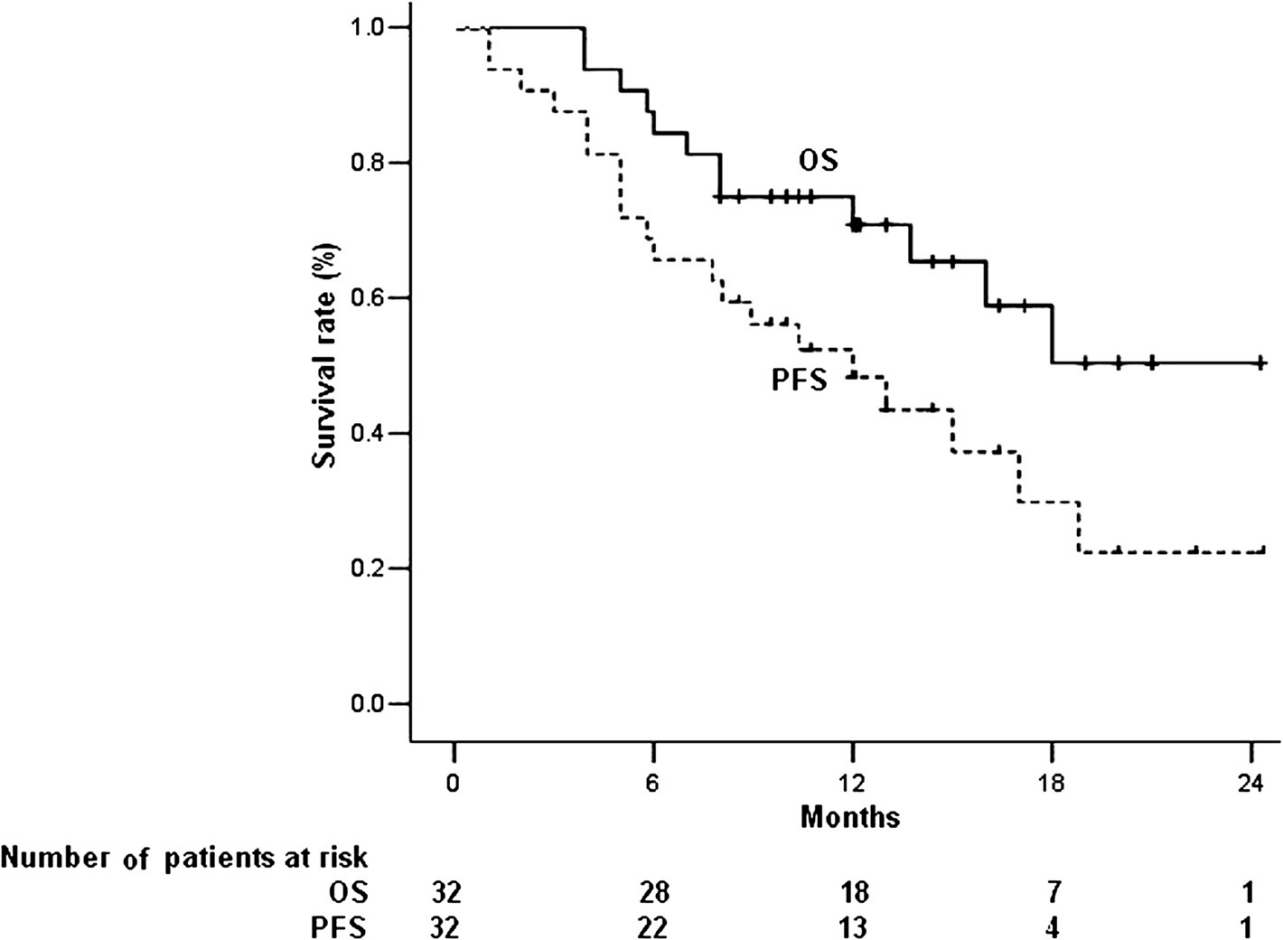
Kaplan-Meier analysis of overall survival (OS) and progression free survival (PFS) of all 32 patients.

**Figure 2:**
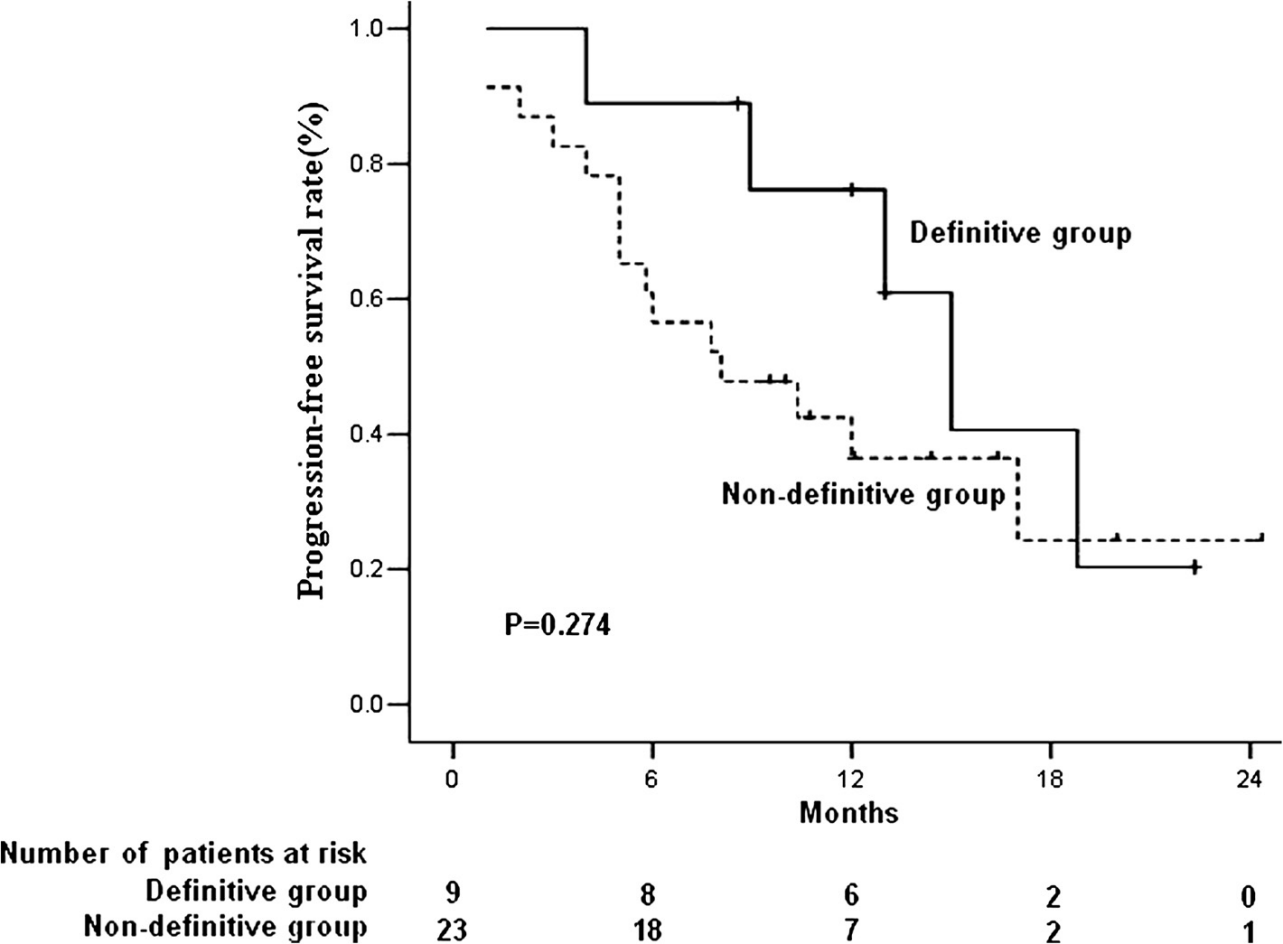
Kaplan-Meier analysis of progression-free survival (PFS) in patients who did and did not receive definitive treatment.

**Figure 3:**
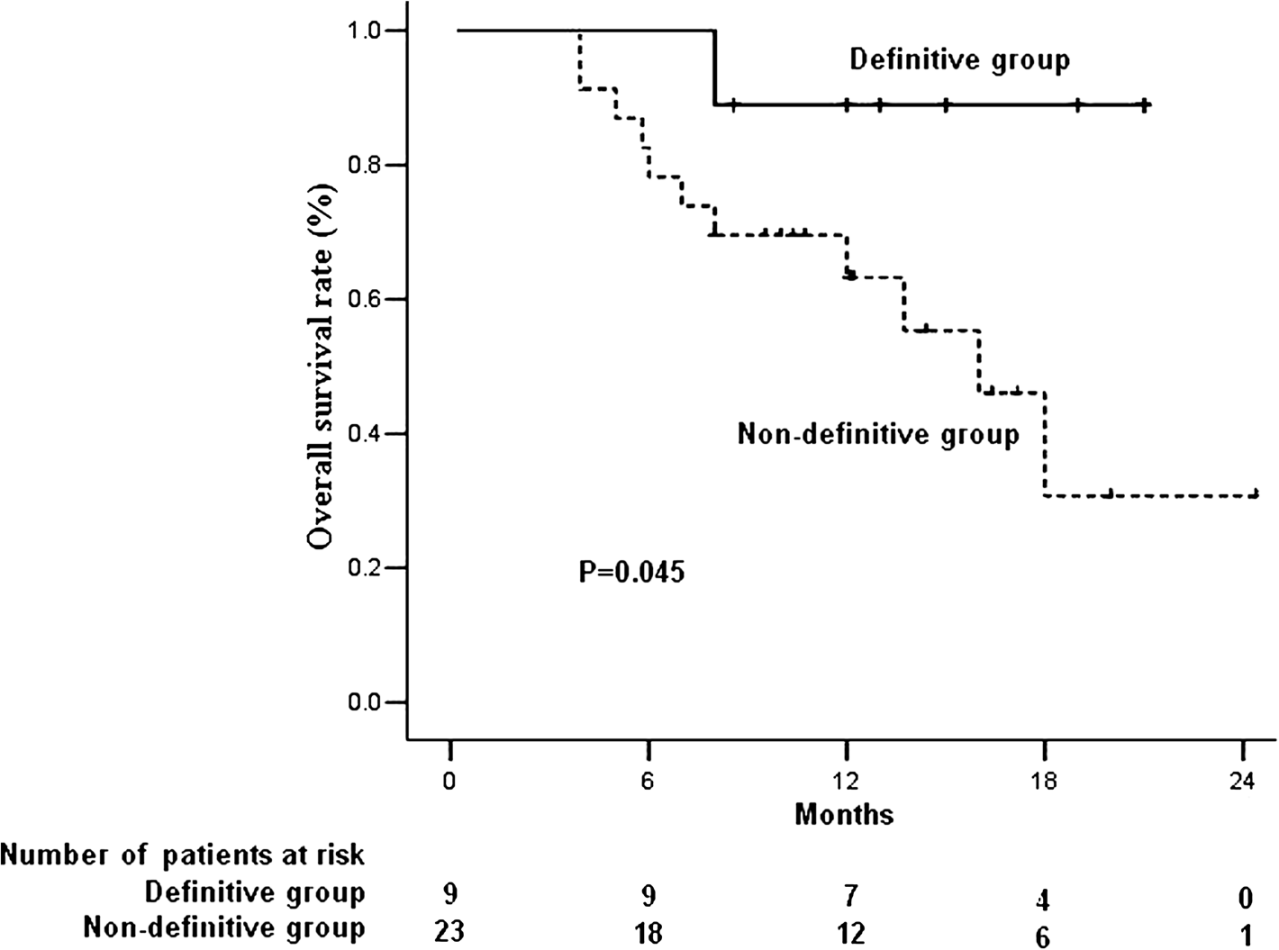
Kaplan-Meier analysis of overall survival (OS) in patients who did and did not receive definitive treatment.

At a median follow-up of 12 months (range, 4–23 months), 5 patients required a decreased chemotherapy dose because of toxicities. Twenty patients experienced tumor progression, including 20 with progression of distant lesions and 2 with progression of local lesions, and 12 patients died of tumor progression. The median OS and PFS of the 32 patients were 17.5 and 12 months (Figure 1). When patients were divided into the 9 who received definitive therapy and the other 23 who did not, there was a significant between group difference in OS (p=0.045; Figure 2), and a non-significant difference in PFS (p=0.274; Figure 3).

### Further analysis of the 9 patients who received definitive therapy

The demographic and clinical features of two groups who received definitive therapy or not are shown in Table 4. No statistic difference was found in all factors between two groups. These 9 patients underwent surgical resection of the primary tumor at a median 13 weeks (range, 11–21 weeks) after CRT, with 5 undergoing APR and 4 undergoing AR. Three patients experienced pCR and downstaging was confirmed in 8 cases. One patient showed disappearance of distant metastases after CRT, whereas the other 8 patients underwent distant resection at a median 15 weeks (range, 14–29 weeks) after the completion of CRT.

**Table 4:** Demographic and clinical characteristics in different groups according to receiving definitive therapy or not

	**Definitive group**	**Non-definitive group**	**P value**
	**(Num)**	**(Num)**	
Gender			
Male	7	21	
Female	2	2	0.557^*^
Age			
Median	57	59	
Range	41-65	35-76	0.765^$^
Distance from anal verge			
Median	5	5	
Range	3-8	3-10	0.649^$^
Initial T stage			
T3	6	13	
T4	3	10	0.238^#^
Initial N stage			
N1	0	3	
N2	9	20	0.541^*^
Initial metastases number			
<=3	2	1	
>3	7	22	0.184^*^
Total	9	23	

^*^ Fisher exact test; ^#^ Chi-square test; ^$^ Non-parameter Mann-Whitney rank-sum test.

At a median follow of 15 months (range, 8–21 months), local-regional and distant recurrences were observed in 0 and 5 patients, respectively. One patient died of liver metastases 8 months after CRT.

## Discussion

In our study, the primary rectal tumor was treated with combined CRT, whereas the distant lesions were treated only with chemotherapy. This chemotherapy regimen, consisting of oxaliplatin plus capecitabine, was expected to improve the overall tumor response, especially for lesions outside the irradiation field. We found that 50% of distant tumors responded to treatment and that this was due solely to weekly chemotherapy, similar to the response to FOLFOX or FOLFIRI in patients with stage IV colorectal cancer [17]. Nine of 32 patients (28.1%) received definitive therapy after CRT and CT, a percentage similar to other studies using FOLFOX [5,6]. Moreover, 30 of these 32 patients experienced progression-free local control after CRT, better than the outcome from chemotherapy alone [17]. Therefore, our preliminary results supported the hypothesis that first-line CRT followed by chemotherapy was superior for primary tumor and non-inferior for distant lesions compared to chemotherapy alone.

Intensified CRT may significantly increase toxicities, which would have a negative impact on subsequent CT. To reduce toxicities, we therefore used the IMRT technique to decrease doses delivered to the surrounding normal organs, including the bowel, bladder and femoral head. For example, use of the IMRT decreased the volume of 45 Gy to the small bowel decreased by more than 64% [18]. Moreover, a comparison of IMRT with 3DCRT in 10 patients found that IMRT had similar target coverage with reduced doses to the small bowel, bladder, pelvic bones and femoral heads [15]. These findings were supported by our results, showing toxicities using IMRT were acceptable and most patients could be treated according to the original CT schedule.

We found that 9 of our 32 patients received definitive treatment, including the removal of all visible masses, and that these patients had a longer PFS and OS. Remove of all visible tumors is critical. Further analysis, however, showed that 5 of these 9 patients experienced distant relapse during follow up. The high distant failure rate was partly due to the initial tumor burden, with most patients having more than 3 distant lesions at initial diagnosis. Total surgical resection after neoadjuvant therapy has been based on the reduction or disappearance of these lesions. However, pCR and cCR rates were not identical, with consistencies of only 50% to 75%. Therefore, recurrence in situ would occur in 40% to 74% of patients whose liver metastases showed a cCR [19,20]. Furthermore, most relapses in our patients occurred after the completion of 6 months of chemotherapy, similar to the results of previous trials [21,22]. Since residual tumor cells may reproduce after the completion of chemotherapy, maintenance chemotherapy may be essential in increasing the disease-free interval in stage IV patients, despite having undergone a “curative surgery”.

## Conclusion

Our preliminary data showed that first-line CRT was effective and tolerable, especially for local control and may be applicable to selected patients with synchronous metastases of rectal cancer. A randomized controlled clinical trial is warranted to compare CRT and CT in patients with stage IV rectal cancer further.

## Competing interests

The authors declare that they have no competing interests.

## Authors’ contributions

ZJ and LP conceived and drafted the manuscript, ZZ drafted and revised the manuscript, and all authors read and approved the final manuscript.

## References

[B1] Manfredi S Lepage C Hatem C Coatmeur O Faivre J Bouvier AM Epidemiology and management of liver metastases from colorectal cancer Ann Surg 2006 244 2 254 259 10.1097/01.sla.0000217629.94941.cf 16858188 PMC1602156

[B2] Van Cutsem E Nordlinger B Adam R European Colorectal Metastases Treatment Group Towards a pan-European consensus on the treatment of patients with colorectal liver metastases Eur J Cancer 2006 42 14 2212 2221 10.1016/j.ejca.2006.04.012 16904315

[B3] Fong Y Fortner J Sun RL Brennan MF Blumgart LH Clinical score for predicting recurrence after hepatic resection for metastatic colorectal cancer: analysis of 1001 consecutive cases Ann Surg 1999 230 3 309 318 discussion 318–21 10.1097/00000658-199909000-00004 10493478 PMC1420876

[B4] Jaeck D Bachellier P Guiguet M Long-term survival following resection of colorectal hepatic metastases. Association Francaise de Chirurgie Br J Surg 1997 84 7 977 980 10.1002/bjs.1800840719 9240140

[B5] Adam R Delvart V Pascal G Rescue surgery for unresectable colorectal liver metastases downstaged by chemotherapy: a model to predict long-term survival Ann Surg 2004 240 4 644 657 discussion 657–8 15383792 10.1097/01.sla.0000141198.92114.f6 PMC1356466

[B6] Alberts SR Horvath WL Sternfeld WC Oxaliplatin, fluorouracil, and leucovorin for patients with unresectable liver-only metastases from colorectal cancer: a North central cancer treatment group phase II study J Clin Oncol 2005 23 36 9243 9249 10.1200/JCO.2005.07.740 16230673

[B7] Kemeny N Management of liver metastases from colorectal cancer Oncology (Williston Park) 2006 20 10 1161 1176 1179; discussion 1179–80, 1185–6 17024869

[B8] Delaunoit T Alberts SR Sargent DJ Chemotherapy permits resection of metastatic colorectal cancer: experience from Intergroup N9741 Ann Oncol 2005 16 3 425 429 10.1093/annonc/mdi092 15677624

[B9] Sauer R Becker H Hohenberger W German Rectal Cancer Study Group Preoperative versus postoperative chemoradiotherapy for rectal cancer N Engl J Med 2004 351 17 1731 1740 10.1056/NEJMoa040694 15496622

[B10] Gerard JP Conroy T Bonnetain F Preoperative radiotherapy with or without concurrent fluorouracil and leucovorin in T3-4 rectal cancers: results of FFCD 9203 J Clin Oncol 2006 24 28 4620 4625 10.1200/JCO.2006.06.7629 17008704

[B11] Bosset JF Collette L Calais G EORTC Radiotherapy Group Trial 22921 Chemotherapy with preoperative radiotherapy in rectal cancer N Engl J Med 2006 355 11 1114 1123 10.1056/NEJMoa060829 16971718

[B12] Roels S Duthoy W Haustermans K Definition and delineation of the clinical target volume for rectal cancer Int J Radiat Oncol Biol Phys 2006 65 4 1129 1142 10.1016/j.ijrobp.2006.02.050 16750329

[B13] Caravatta L Padula GD Picardi V Concomitant boost radiotherapy and multidrug chemotherapy in the neoadjuvant treatment of locally advanced rectal cancer: results of a phase II study Acta Oncol 2011 50 8 1151 1157 10.3109/0284186X.2011.582880 21851185

[B14] Therasse P Arbuck SG Eisenhauer EA New guidelines to evaluate the response to treatment in solid tumors. European Organization for Research and Treatment of Cancer, National Cancer Institute of the United States, National Cancer Institute of Canada J Natl Cancer Inst 2000 92 3 205 216 10.1093/jnci/92.3.205 10655437

[B15] Mok H Crane CH Palmer MB Intensity modulated radiation therapy (IMRT): differences in target volumes and improvement in clinically relevant doses to small bowel in rectal carcinoma Radiat Oncol 2011 6 63 10.1186/1748-717X-6-63 21651775 PMC3121606

[B16] Kaplan EL Meier P Non parametric estimation from incomplete observations J Am Stat Assoc 1958 53 457 481 10.1080/01621459.1958.10501452

[B17] Tournigand C Andre T Achille E FOLFIRI followed by FOLFOX6 or the reverse sequence in advanced colorectal cancer: a randomized GERCOR study J Clin Oncol 2004 22 2 229 237 14657227 10.1200/JCO.2004.05.113

[B18] Guerrero Urbano MT Henrys AJ Adams EJ Intensity-modulated radiotherapy in patients with locally advanced rectal cancer reduces volume of bowel treated to high dose levels Int J Radiat Oncol Biol Phys 2006 65 3 907 916 10.1016/j.ijrobp.2005.12.056 16751073

[B19] Benoist S Brouquet A Penna C Complete response of colorectal liver metastases after chemotherapy: does it mean cure? J Clin Oncol 2006 24 24 3939 3945 10.1200/JCO.2006.05.8727 16921046

[B20] Tanaka K Takakura H Takeda K Matsuo K Nagano Y Endo I Importance of complete pathologic response to prehepatectomy chemotherapy in treating colorectal cancer metastases Ann Surg 2009 250 6 935 942 10.1097/SLA.0b013e3181b0c6e4 19953712

[B21] Portier G Elias D Bouche O Multicenter randomized trial of adjuvant fluorouracil and folinic acid compared with surgery alone after resection of colorectal liver metastases: FFCD ACHBTH AURC 9002 trial J Clin Oncol 2006 24 31 4976 4982 10.1200/JCO.2006.06.8353 17075115

[B22] Nordlinger B Sorbye H Glimelius B EORTC Gastro-Intestinal Tract Cancer Group; Cancer Research UK; Arbeitsgruppe Lebermetastasen und-tumoren in der Chirurgischen Arbeitsgemeinschaft Onkologie (ALM-CAO); Australasian Gastro-Intestinal Trials Group (AGITG); Fédération Francophone de Cancérologie Digestive (FFCD) Perioperative chemotherapy with FOLFOX4 and surgery versus surgery alone for resectable liver metastases from colorectal cancer (EORTC Intergroup trial 40983): a randomised controlled trial Lancet 2008 371 9617 1007 1016 10.1016/S0140-6736(08)60455-9 18358928 PMC2277487

